# Allocation of Scarce Resources in a Pandemic: A Systematic Review of US State Crisis Standards of Care Documents

**DOI:** 10.1017/dmp.2020.101

**Published:** 2020-04-16

**Authors:** Douglas Romney, Hannah Fox, Stephanie Carlson, Daniel Bachmann, Donal O’Mathuna, Nicholas Kman

**Affiliations:** OSU Wexner Medical Center Department of Emergency Medicine, Columbus, Ohio

**Keywords:** allocation of resources, coronavirus, disaster planning, pandemics

## Abstract

The aim of this systematic review was to locate and analyze United States state crisis standards of care (CSC) documents to determine their prevalence and quality. Following PRISMA guidelines, Google search for “allocation of scarce resources” and “crisis standards of care (CSC)” for each state. We analyzed the plans based on the 2009 Institute of Medicine (IOM) report, which provided guidance for establishing CSC for use in disaster situations, as well as the 2014 CHEST consensus statement’s 11 core topic areas. The search yielded 42 state documents, and we excluded 11 that were not CSC plans. Of the 31 included plans, 13 plans were written for an “all hazards” approach, while 18 were pandemic influenza specific. Eighteen had strong ethical grounding. Twenty-one plans had integrated and ongoing community and provider engagement, education, and communication. Twenty-two had assurances regarding legal authority and environment. Sixteen plans had clear indicators, triggers, and lines of responsibility. Finally, 28 had evidence-based clinical processes and operations. Five plans contained all 5 IOM elements: Arizona, Colorado, Minnesota, Nevada, and Vermont. Colorado and Minnesota have all hazards documents and processes for both adult and pediatric populations and could be considered exemplars for other states.

Since the terrorist attacks of 2001, the hurricane season of 2005, and the Ebola and COVID-19 outbreaks, significant resources have been used in the United States to improve the processes behind care provided in a disaster or pandemic. Federal efforts to formalize disaster response have been attempted at the state and local level, often with the assistance of funding from governmental and private agencies. The term “crisis standards of care” (CSC) describes a substantial change in health-care operations and the level of care that can be delivered in a public health emergency, such as a pandemic or natural disaster.^1^ In CSC, hospitals would use adapted spaces with staff and supplies that are not consistent with usual standards of care. Extraordinary measures would be necessary to provide the best possible care to patients given the circumstances and resources available. In this type of disaster, focus shifts from individual patient-centered care to population-centered outcomes.^1^

The literature overwhelmingly recommends preparing for these catastrophes.^2^ Without this proactive planning, resource management will be chaotic and inequitable, as we have seen with COVID-19. There are a variety of recommendations for specific topics available, such as disaster training for health-care professionals^3^ and allocation of critical care resources.^2,4^ These documents are realistic and frank about the change in level and type of care provided due to disaster limitations. Therefore, ethical implications are frequently discussed, in an effort to ensure that responding physicians have a framework to support the difficult decisions they may have to make.^5,6^

At the state level, efforts have been made to develop formalized protocols for the allocation of scarce resources in the event of a pandemic or disaster. These documents are prescriptive in nature and are intended for use at multiple levels from the state down to the provider. They are typically developed by committees of topic experts, stakeholders, and community members, who rely on precedent from other states, guidance from nonprofit and academic organizations, as well as personal experience. In 2014, we (N.K., D.R.) were invited to participate in the state of Ohio’s development of a CSC document. Although the document remains in draft form at the state government level, this experience led to knowledge of these state documents and the processes needed to develop them.

The state guiding documents are heterogeneous in content, length, and topic matter. In an attempt to decrease this variability and encourage more states to draft plans, 2 leading nonprofit nongovernmental medical organizations each outlined characteristics that they believed to be necessary in a state CSC plan. In the midst of the 2009 H1N1 pandemic, the Assistant Secretary for Preparedness and Response (ASPR) asked the Institute of Medicine (IOM), now called the National Academy of Medicine, to convene a committee of experts to develop guidance that health officials could use to establish and implement standards of care during disasters. They identified 5 key elements for state CSC protocols (Table 1),^1,7^ while in 2014, the CHEST Task Force Executive Committee identified 11 core topic areas (Table 2).^8^ We used these criteria to evaluate state CSC plans. Our aim was not to criticize any plan, but to identify exemplars that can guide states and institutions as they develop their plans, especially if needed urgently.

**TABLE 1:** 2009 IOM Key Elements for State Crisis Standards of Care Protocols by State (ref 2)

Key Element	No. of State Plans Containing Element
Strong ethical grounding	18
Integrated and ongoing community and provider engagement, education, and communication	21
Assurances regarding legal authority and environment	22
Clear indicators, triggers, and lines of responsibility	16
Evidence-based clinical processes and operations	28

**TABLE 2:** CHEST Guidelines Core Topic Areas Regarding the Provision of Care to Critically Ill or Injured Patients From Pandemics or Disasters by State

Core Topic Area	No. of State Plans Containing Core Topic Area
Business and continuity of operations	19
Education	14
Engagement	15
Ethics/culture	17
Mobilization and evacuation	3
Systems planning, coordination, and communication	19
Policy/legal	21
Triage	18
Resource-poor settings	16
Special populations	18
Surge capacity	20

## METHODS

**FIGURE 1:**
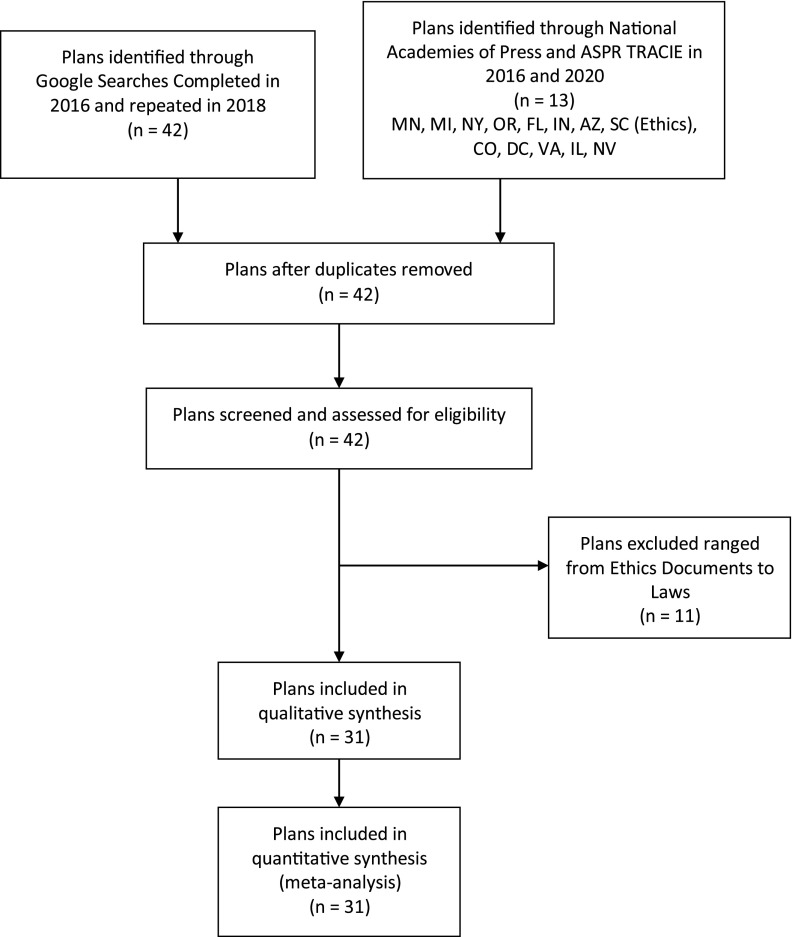
PRISMA Diagram

Following PRIMSA guidelines (Figure 1), US State CSC plans were initially identified using a standard Google search using the following queries: “allocation of scarce resources <STATE>”, “<STATE> Pandemic Plan”, and “crisis standards of care <STATE>” for all 50 states and the District of Columbia. The first 2 pages of Google search results were screened for CSC documents or references to a state document.

This search was performed originally in March of 2016. The search was repeated in August of 2018. Subsequently, the reference documents from the National Academies of Press and ASPR TRACIE were searched for state plans, initially performed in March of 2016 and repeated in 2019 and 2020. The state plans found on the ASPR search were also identified through Google. Many of the documents are in draft form. Searches were continually repeated as these documents change and hosting websites often change as well. Furthermore, some of the documents referenced may have been removed from the Web.

Forty-one states and the District of Columbia were found to have discoverable documents. One state was found to have reference to a document not immediately found in Google search results, but which was already available to researchers from efforts in the development of a state plan for Ohio. Among the 42 plans, we immediately excluded 11 plans that were not actual CSC documents. The documents that were excluded ranged from a collection of emergency management laws to ethical recommendations only. For the states that had separate types of plans (Utah Pandemic and Utah Burn), we counted these once only. The remaining 31 plans were systematically reviewed for critical elements contained in the 2009 IOM letter and for the 11 elements identified in the 2014 CHEST consensus statement. These findings are outlined in Tables 1 and 2.

## RESULTS

Overall, there were 5 plans that contained all 5 IOM elements: Arizona, Colorado, Minnesota, Nevada, and Vermont. Arizona, Colorado, Nevada, and Vermont adopted an all-hazards approach. Minnesota has several documents including a Pandemic Influenza Plan supplemented by a separately identified Scarce Resource Strategies section. In fact, this section is included in the Colorado, Nevada, and Vermont plans.

Eighteen of the state plans had strong ethical grounding. Almost all plans mentioned ethics, with many referring to other guidelines like those produced by the University of Toronto, New York, or Minnesota.^9-11^ The tragic dilemmas and intense moral distress, exemplified during the COVID-19 pandemic, have produced further ethics guidance.^12,13^ Among the state plans that explicitly developed ethical grounding, Minnesota and Arizona were exemplary. In 2006, Minnesota commissioned a detailed study of relevant ethical issues. This was published in 2010 and included the results of public engagement exercises on ethics held around Minnesota, their detailed ethical reasoning, and a list of recommendations for ethical decision-making when rationing different types of interventions (eg, antiviral medications, masks, ventilators, etc.). This detailed and thorough report was used by several other state plans.^11^ The Minnesota Pandemic Ethics Project continues to produce new resources.^11^ Arizona’s 2015 plan had ethics as foundational to the whole report, with the additional goal of ensuring that pandemic responses are compassionate.^14^ Their working group also developed a Crisis Standards of Care Emergency Code of Ethics for Arizona, and provided some ethical decision-making guidance. Nevada’s CSC plan (2017) had many similarities to Arizona’s, although did not address issues in the same level of detail.^15^ Colorado’s plan (2009) had a general discussion of ethics.^16^ Michigan’s plan (2012) focused on ethics throughout its discussions, and provided excellent descriptions of the key ethical principles.^17^ Louisiana’s plan (2014) was explicitly based on ethical principles that were clearly defined.^18^ Finally, Indiana’s plan (2014) thoroughly examined ethical issues with triage and scare resources, but did not explore any other ethical issues.^19^

Twenty-one of the state plans had integrated and ongoing community and provider engagement, education, and communication. As an exemplar, Arizona’s plan had a robust preincident development strategy. They used workgroups made up of stakeholders from public health, health care, law, ethics, and emergency management. These groups prepared different sections, including a dedicated public engagement campaign. This public engagement campaign included public meetings, as well as an online feedback tool to assess public opinion and garner support. Communication during an incident was addressed in 1 section, with multiple communication systems outlined with purposes, administrators, and target audiences. Finally, it had sections that addressed postincident behavioral health needs of the general public and medical professionals, as well as the continuation of care for those with existing serious mental illness.

Twenty-two of the reviewed plans had assurances regarding legal authority and environment. Of the 5 plans highlighted by our review, Arizona had the most comprehensive inclusion of legal considerations. Based on the IOM CSC Framework of 2012, the Arizona plan addressed the following legal issues: personnel, access to treatment, coordination of health services, patient’s interest, resource allocation, liability, reimbursement, and inter-jurisdictional cooperation.^14^ The second exemplar state CSC plan regarding legal issues was Nevada.^15^ This plan covered in sufficient depth all the aspects of the CHEST paper except for consideration of evacuation and/or sheltering-in-place. It went further in some areas to include recommendations for worker/volunteer reimbursement as well as guidelines for parties that fail to meet the CSC specifications. A third state with comprehensive legal guidelines was Colorado.^16^

A few more plans had legal sections worthy of being highlighted. Connecticut’s CSC plan contains a well-organized table of the state and federal statutes associated with CSC and examples of their applicability during a disaster.^20^ North Dakota had a brief but functional section on legal aspects of CSC with good breadth albeit less depth.^21^ Finally, a regional plan from Georgia has a legal section comparable in quality to the 3 exemplars listed above (Arizona, Nevada, Colorado). It was excluded from the final list as it is not a “state” plan, but it is still a good model for legal considerations.

Sixteen of the plans had clear indicators, triggers, and lines of responsibility. In many of the state plans, this trigger was a formal declaration of emergency or disaster by the Governor or an executive health committee. Indicators are measures or predictors of changes in demand and/or resource availability; triggers are decision points. Indicators and triggers guide transitions along the continuum of care, from conventional to contingency to crisis and then the return to conventional.^22^ Furthermore, the IOM Toolkit defines a crisis care trigger as the point at which the scarcity of resources requires a transition from contingency care to crisis care, implemented within and across the emergency response system. This is the transition point at which resource allocation strategies focus on the community rather than the individual. Table 3 shows examples of indicators and triggers used for implementation of CSC plans (not state specific).

**TABLE 3:** Examples of Indicators and Triggers Used for Implementation of CSC Plan

Standard of Care Level	Conventional Standards of Care Usual Care, Normal Operating Conditions	Contingency Standards of Care Functionally Equivalent Care	CSC Extreme Operating Conditions
Space triggers	Usual patient care space fully utilized	Patient care areas re-purposed (PACU, monitored units for ICU-level care). Doubling of patient rooms; abnormally high percentage of hospitals on divert for EMS	Facility damaged/unsafe or non-patient care areas (classrooms, etc.) used for patient care (alternate care sites)
Staff triggers	Usual staff called in and utilized	Staff extension (brief deferrals of nonemergent service, supervision of broader group of patients, change in responsibilities, documentation, etc.)	Trained staff unavailable or unable to adequately care for volume of patients even with extension techniques; marked increase in staff or school absenteeism (2 specifying 20-30% or >30% thresholds)
Supply triggers	Cached and usual supplies used	Conservation, adaptation, and substitution of supplies with occasional re-use of select supplies	Critical supplies lacking, possible reallocation of life-sustaining resources (ventilators, beds, blood products, antivirals, PPE, etc.)
Indicators	Full utilization of space, staff, and supplies is a potential indicator for contingency standards of care	Maximization of contingency standards of care is a potential indicator for CSC	

Abbreviations: CSC, crisis standards of care; EMS, emergency medica; services; ICU, intensive care unit; PACU, post-anesthesia care unit; PPE, personal protective equipment.

Colorado has a 10-step process for activation of the CSC plan. There are local triggers that inform points for declaring local disasters and/or requesting Governor’s disaster declaration and implementation of CSC. They additionally discuss state indicators. In Minnesota’s plans, The Minnesota Division of Homeland Security and Emergency Management (HSEM) is the lead coordinating agency during any statewide emergency, including a pandemic. The Minnesota Department of Health will work closely with HSEM as well as with other state and local partners during a pandemic and will be the lead technical agency in the state during pandemic phases. Finally, Arizona has well-defined indicators, triggers, and authority in their plan that might inform other plans. Arizona specifically mentions supplies, staff, and space considerations, including personal protective equipment (PPE) supplies (e.g., N95 masks), medications (antivirals, antibiotics, analgesics, paralytics), as well as outpatient, inpatient, and alternate care sites. Staff illness, family obligations, or reluctance to report are also mentioned as contributing to difficulty with adequate staffing.^14^

Twenty-eight of the plans had evidence-based clinical processes and operations. Most use an evidence-based scoring system for ventilator and ICU usage in pandemics. Several of the state plans designate prehospital triage systems. Most plans state that objective criteria should be used in triage decisions. Colorado and Minnesota, among others, consider pediatric populations specifically. Many of the plans reference Utah,^23^ which has a very easy to follow triage process that uses Modified Sequential Organ Failure Assessment (MSOFA) as a part of its allocation process.^24^

## DISCUSSION

In 2009, the IOM committee on guidance for establishing standards of care for use in disaster situations published a report providing guidance for establishing CSC plans for use in disaster situations. Many states had either developed or were in the process of developing state plans to address this need. To complement the IOM’s work, we performed a systematic review to assess the publicly accessible state CSC documents.

Based on our analysis, several conclusions may be reached regarding the current preparation for disaster scenarios at the state level. It may be surprising to many readers that nearly 20 states provide no explicit guidance for health-care entities or workers functioning during times of pandemic or crisis. In terms of the IOM elements, Arizona, Colorado, Minnesota, Vermont (draft),^25^ and Nevada all are comprehensive plans. Each of these 5 plans have similar ethical sections, based on core ethical principles such as justice and fairness, duty to care, and proportionality. They have comprehensive and detailed legal frameworks, including specific federal and state laws. They have well-defined activation and triggers, identifying specific individuals who may activate the plan and when it should be activated. Each plan has considerations for special populations, specifically pediatric considerations.

Beyond the IOM elements, there are some differentiating factors between these IOM complete plans. First, they vary in their length. Colorado has a manageable length of 88 pages, enough to be thorough but not prohibitive for the average reader. One particular area that makes Colorado an exemplar plan is in the decision for critical care. As we are seeing with the COVID-19 pandemic in Italy, supplies such as ventilators can be a scarce resource in need of guidelines for allocation. Colorado’s plan has specific inclusion and exclusion criteria for critical care, as well as patient prioritization. These exclusion criteria include end-stage organ failure and incurable metastatic disease, among others. Meanwhile, while Arizona and Nevada do have clear inclusion criteria that are similar to Colorado’s, there are no clear exclusion criteria. For example, Nevada’s plan states that the Sequential Organ Failure Assessment (SOFA) score may be used to “prioritize admission for ICU-level care or to reallocate scarce medical resources.” In contrast, Colorado’s plan specifies MSOFA scores that should lead to withdrawal of care. “If a patient has a MSOFA greater than 8 for greater than 5 days, and with flat or rising trend or if a patient ever has a MSOFA score of 15 or higher or any other exclusion criteria, they should be considered for withdrawal from ongoing critical care”.^16^ Although there is some controversy regarding the use of SOFA scores in exclusion criteria, new literature suggests using the SOFA or the Pediatric Logistic Organ Dysfunction 2 (PELOD-2) scoring systems to predict mortality over the short term.^26^

Many of the documents focus on the provision of critical care in a pandemic or disaster, including the allocation of ventilators. Indiana^19^ and Florida^27^ would be examples of plans that do not have sections on indicators, triggers, and lines of authority but instead move quickly into addressing triage and how life-saving treatments, namely pressors and ventilators, would be allocated in a disaster. New York has a ventilator-specific plan that contains adult guidelines, pediatric guidelines, neonatal guidelines, and legal considerations.^10^ Another issue that is addressed by the New York plan is the topic of using age as an exclusion factor. Their draft guidelines recommend that “advanced age should not be a factor that prevents a patient from being eligible for ventilator therapy.” Age is an issue that is avoided by most state plans, but recent literature suggests that it may be used as a tie-breaker in patients who have equivalent prognoses.^26^ This is referred to as stage of life-related (life-cycle) and suggests that children and adults under the age of 50 be given the highest priority for a ventilator in the event of a tie. As age increases in this model, priority decreases with lowest priority given to adults over the age of 85.

Of the states that do have plans in place, only 13 of them have documents outlining an “all hazards” approach with the remaining state plans limited to specific disaster scenarios, typically pandemic influenza. While these guidelines may be adapted by an individual user or organization to respond to other disaster situations, the legal protections and guidance provided therein would be implied only and not specifically codified in the event of, for example, a chemical or radiological event. As we are seeing in states heavily impacted by COVID-19 surges, communication is limited and societal structure may be fragmented, making it a particularly poor situation for the development and dissemination of new guidelines or guidance documents.

The 2009 IOM guidance called on states to develop consistent CSC protocols with the 5 key elements described above.^7^ More recently, the Office of the Assistant Secretary for Preparedness and Response (ASPR) issued the 2017-2022 Health Care Preparedness and Response Capabilities document that outlines the nation’s disaster preparedness and response.^28^ ASPR’s Hospital Preparedness Program (HPP), which provides federal funding for disaster response, also necessitates planning for a move to CSC. We were able to identify 13 plans on ASPR’s website, with Minnesota and Michigan listed under the “Must Read” section under Crisis Standards of Care. Despite this level of guidance, massive heterogeneity exists in the 31 state plans we analyzed. Additionally, as we are seeing with the COVID-19 pandemic, much of the planning, obtaining, and allocating of resources is left to the states, rather than being federally mandated or required.

In summary, there is inconsistency in the application of the guidance to states on the allocation of scarce resources during a time of crisis. At the state level, 31 states have a complete CSC document. Thirteen of these documents embrace an all hazards approach, while 18 are pandemic specific. The good news is that many of these plans are publicly available using a simple Google search, and many of the plans do address critical elements as recommended by the IOM. Furthermore, professional societies, such as the American College of Chest Physicians (ACCP) and the American College of Emergency Physicians (ACEP), are pushing this discussion forward.^29,30^ In sum, a more transparent identification of exemplar state plans, including a toolkit drawing from these state plans, may assist policy-makers in states that have not yet finished a CSC document.

## LIMITATIONS

No central public repository exists for state plans related to CSC and allocation of scarce resources. Although we repeated our search several times since this project started, it is possible that our approach did not identify every state plan. We identified 31 CSC state plans. In a recent article in the lay press, Dr. John Hick, who helped with the original ASPR-funded CSC project, identified 36 state plans.^31^ Our search used the terms, “allocation of scarce resources <STATE>”, “<STATE> Pandemic Plan” and “crisis standards of care <STATE>” which are commonly accepted in this field. It is possible that other search terms would have led to the discovery of other state plans. Furthermore, although our search strategy did capture every document that was previously known to us, it is possible that some state plans are intentionally less accessible to the public and not readily captured using a Google search. We were only able to find 13 state plans directly through ASPR.

Second, our objective was to conduct a systematic review of existing state plans related to the allocation of scarce resources and identify an exemplar plan that can be used to guide states in drafting these. That said, there is no “gold standard” document against which to measure these plans. Our review of the current literature on this topic identified the IOM and CHEST guidelines as the closest to a “gold standard” that exists, and both were included for a more robust analysis of the state plans.

Finally, many plans did not adopt an all hazards approach and were designed to address a specific disaster or pandemic scenario. As such, many elements recommended by the IOM and CHEST may not have been included merely because of the limited scope of the state plan. On the other hand, the inclusion of plans that were not specifically CSC documents may have led to an overestimation of the number of states that have usable documents in the event of a disaster.

## CONCLUSIONS

There may be inadequate guidance to inform providers and policy-makers about the most effective strategies for allocating scarce resources during a time of crisis. Many states currently lack a publicly available CSC plan in any form. Of 31 state CSC documents identified here, 18 plans embrace a pandemic approach. Explicit standards are not mandated at the federal level to guide these plans, which are heterogeneous in their approach, content, and length. Five plans contained all 5 IOM elements: Arizona, Colorado, Minnesota, Nevada, and Vermont. Colorado and Minnesota have all hazards documents and processes for both adult and pediatric populations and could be considered exemplars for other states.
